# Workplace policies and practices promoting physical activity across England: What is commonly used and what works?

**DOI:** 10.1108/IJWHM-01-2017-0004

**Published:** 2017-10-02

**Authors:** Emily Caitlin Lily Knox, Hayley Musson, Emma J. Adams

**Affiliations:** 1 Queens Medical Centre, University of Nottingham, Nottingham, UK; 2 National Centre for Sport and Exercise Medicine, School of Sport, Exercise and Health Sciences, Loughborough University, Loughborough, UK

**Keywords:** Workplace, Environment, Worksite, Public health, Physical activity, Promotion, Policies

## Abstract

**Purpose:**

Many adults fail to achieve sufficient moderate-to-vigorous physical activity (MVPA). The purpose of this paper is to understand how workplaces most effectively promote physical activity for the benefit of public health.

**Design/methodology/approach:**

Data were collected via two online surveys. First, 3,360 adults employed at 308 workplaces across England self-reported their MVPA, activity status at work and frequency of journeys made through active commuting. From this sample, 588 participants reported on the policies and practices used in their workplace to promote physical activity. Factor and cluster analysis identified common practice. Regression models examined the association between the workplace factors and engagement in physical activity behaviours.

**Findings:**

Five factors emerged: targeting active travel, availability of information about physical activity outside the workplace, facilities and onsite opportunities, sedentary behaviour, and information about physical activity within the workplace. Further, five clusters were identified to illustrate how the factors are typically being utilised by workplaces across England. Commonly used practices related to promoting active travel, reducing sedentary behaviour and the provision of information but these practices were not associated with meeting MVPA guidelines. The provision of facilities and onsite exercise classes was associated with the most positive physical activity behaviour outcomes; however, these structures were rarely evident in workplaces.

**Originality/value:**

Previous research has identified a number of efficacious actions for promoting physical activity in the workplace, however, research investigating which of these are likely to be acceptable to worksites is limited. The present study is the first to combine these two important aspects. Five common profiles of promoting physical activity in worksites across England were identified and related to physical activity outcomes. Guidance is given to workplace managers to enable them to maximise the resources they have for the greatest gains in employee health. Where feasible, facilities, and classes should be provided to achieve the most positive outcomes.

## Background

Many adults in England are insufficiently active (Department of Health Physical Activity Health Improvement and Protection, 2011). Engaging in sufficient levels of physical activity is associated with benefits to the health of individuals (Lee *et al.*, 2012). A healthy active workforce has been associated with increased productivity and reduced absenteeism (National Institute for Health and Care Excellence, 2008; Robroek *et al.*, 2011). Further, while evidence around workplace physical activity programs is equivocal, well-designed programs do have the potential to improve employee health and productivity (Pereira *et al.*, 2015).

Adults spend around 60 per cent of their waking hours at work (Peersman *et al.*, 1998) making it a “hub” from which large groups with existing social networks can be reached by efforts to promote healthful behaviours (Robroek *et al.*, 2009). Efforts to promote physical activity within the workplace setting have produced modest positive changes in both employee physical activity and health (Hutchinson and Wilson, 2012). In 2008, the Black Report reviewed the health of the working age population in the UK (Cross-Government Health, Work and Well-being Programme, 2008a,  b). It concluded that employer’s needed to provide clear guidance on how to improve health in the workplace, greater support should be directed towards small- and medium-sized enterprises, current measures such as the sick note were not working, and provision should be extended. Despite this, the applicability of evidence for workplace physical activity programs to many workplaces is not obvious and the advice given is often weak.

A recent review has suggested that physical activity promotion strategies at the organisational level (i.e. within workplaces, schools, etc.) may be more sustainable than individual level strategies (Barr-Anderson *et al.*, 2011). A number of organisations such as the Centre for Disease Control (www.cdc.gov/workplacehealthpromotion/implementation/topics/physical-activity.html) and the National Institute for Health and Care Excellence (2008) have released guides for implementing physical activity policies and programmes within the workplace highlighting both individual and organisational approaches.

Interventions introduced into worksites can target a wide variety of physical activities. For instance, it seems likely that interventions targeting reduced sedentary behaviour (Shrestha *et al.*, 2016), active transport (Petrunoff *et al.*, 2016) and even team sport (Brinkley *et al.*, 2016) could positively influence individual and group physical activity behaviour. However, systematic reviews of randomised-controlled trials provide strong evidence of effectiveness they do not always consider feasibility and acceptability to the worksite (Fitzgerald *et al.*, 2016). Further, it is not known to what extent workplaces prioritise the promotion of physical activity. In addition, the provision of environmental and policy-based support in workplaces, and their impact on physical activity levels, is not well understood. This knowledge would be useful to ensure the interventions with evidence of effectiveness but also evidence of acceptability to worksites are promoted. Some work has been conducted in this area. Crespo *et al.* (2011) asked workers across three regions of the USA to report the presence of nine physical activity promoting environment and policy strategies (e.g. presence of lockers, showers, facilities, etc.) in their workplace and found the number of strategies to be positively related to recreational physical activity. However, this was not a comprehensive study and nor has any equivalent research been conducted in the UK.

It would also be useful to identify which interventions with evidence of effectiveness are most acceptable to worksites as it may be financially prohibitive for employers to introduce many new measures especially when many employees may refrain from taking part and positive outcomes are not guaranteed (Robroek *et al.*, 2009). It would therefore be of benefit to know which policies and practices are typically being used by organisations to promote physical activity and which ones offer the greatest likelihood of success in terms of increasing the physical activity of employees. This will enable more effective use of resources when it comes to promoting physical activity within the workplace.

The aim of this study was to identify common practices and policies employed by workplaces in England to promote physical activity to employees. A further aim was to identify the association of these commonly employed strategies on active commuting, workplace physical activity and sedentary behaviour and meeting moderate-to-vigorous physical activity (MVPA) guidelines.

## Methods

The research design was cross-sectional and involved two online surveys. Ethical approval was received from the host institutions ethics committee. The present study reports on data collected through the CSPN Workplace Challenge. The CSPN Workplace Challenge is a national campaign which provides information and opportunities for individuals to engage in sport through their local sports partnerships. Adults from any workplace across England are able to sign up to the Challenge. After registering with the project website, participants are invited to complete a baseline survey and then encouraged to log their engagement using an online tool (see www.workplacechallenge.org.uk/national-challenge for more information). In total, 11,074 adults working in England completed the online survey between 1 October 2013 and 30 September 2014. The present study uses the following self-reported items from the Workplace Challenge Survey.

### Demographics

Participants self-reported their gender, age, ethnicity, education, job type, workplace postcode and health status.

### Overall MVPA

The short form of the International Physical Activity Questionnaire was used (Craig *et al.*, 2003). Participants reported the minutes they engaged each week in moderate and vigorous physical activities and in walking. From this it was then calculated whether participants met MVPA guidelines of at least 150 minutes a week.

### Activity status at work

Questions were taken from the European Prospective Investigation in Cancer and Nutrition questionnaire (Medical Research Council, Cambridge). Participants responded to the stem question “is your work mostly?” by selecting from one of four options: “sedentary i.e. you spend most of your time sitting”; “manual i.e. involves some physical effort including handling of heavy objects and use of tools”; “standing i.e. you spend most of your time standing or walking, but your work does not require intense physical effort”; and “heavy manual i.e. this implies very vigorous physical activity including handling of very heavy objects”.

### Active commuting

Questions were adapted from the Transport and Physical Activity Questionnaire (Adams *et al.*, 2014). Participants reported the number of days a week that they jogged, walked and cycled to and from work. These variables were summed to give the overall number of journeys actively commuted.

From the sample completing the Workplace Challenge Survey, 2,004 participants (18.1 per cent) stated that they had a responsibility to promote health within their workplace. A follow-up Worksite Health Promotion Survey was sent to these individuals in July 2014 in order to capture data regarding the strategies used to promote physical activity within their workplace.

The Worksite Health Promotion survey was completed by 588 participants (29.3 per cent). It contained 50 self-report items based on the ecological model and research by Hipp *et al.* (2015), including promotions and programs, organisational policies and practices, internal physical environment, internal social environment, and external environment. Respondents were asked to select by means of tick-box, each of the 50 possible policies, facilities, support structures, communications, actions and promotions used by the workforce at their workplace to promote physical activity (see factor analysis results).

### Statistical analyses

There were two stages of statistical analyses.

#### Stage 1: factor analysis and cluster analysis

Stage 1 used only the Worksite Health Promotion Survey. A factor analysis was first conducted to identify common factors described by the 50 items. Initial exploratory analysis with examination of the Scree plot identified the number of factors in the data. Subsequent analysis using the varimax method with Kaiser normalisation ascertained factor loading of the 50 items. Next, a hierarchical cluster analysis was performed using Ward’s method and Euclidean distance (Milligan and Martha, 1987) to profile the workplaces according to their inclusion of the factors identified through factor analysis. The number of clusters was decided based upon observed changes in the agglomeration schedule coefficient. These clusters were then verified using K-means analysis. This was done by running K-means analysis with the number of clusters set according to the outcome of the hierarchical method and checking for similarities of cluster membership. Further, we ran K-means analysis with the number of clusters set within a range of one around that identified by the hierarchical method and graphically examined the data to confirm whether optimal clustering had been achieved. As the factors contained different numbers of items, variables were standardised by dividing by the range (Milligan and Martha, 1987). Mahalanobis *D*^2^ was used to check for multivariate outliers. Cluster characteristics are described following examination of standardized *z*-scores of respective values for the five factors.

#### Stage 2: association of workplace factors with physical activity variables

Stage 2 used data from the Worksite Health Promotion Survey alongside data from the Workplace Challenge Survey. Multinomial logistic regression examined the association between the workplace factors identified through factor analysis and physical activity status and workplace physical activity. Linear regression analyses examined associations between the five workplace factors and the frequency of active commuting. All regressions controlled for gender, age, ethnicity, education, health status and job type.

## Results

### Participant and workplace characteristics

Of the 588 respondents to the Worksite Health Promotion Survey, 485 provided valid postcodes for their workplace, of which 308 were unique. From the Workplace Challenge Survey, 3,360 respondents reported working in one of these 308 workplaces and so provided the physical activity variables for stage 2 analyses. Respondents to the Workplace Challenge Survey were 66.8 per cent female, 95.4 per cent White British and had an average age of 39.5 years (Table I). Respondents to the Worksite Health Promotion Survey were from workplaces which were 36.4 per cent statutory organisations, 73 per cent multi-site, 65.5 per cent based in a city or town and 61.9 per cent employed more than 251 people (Table II).

### Factor and cluster analysis

**Figure 1:**
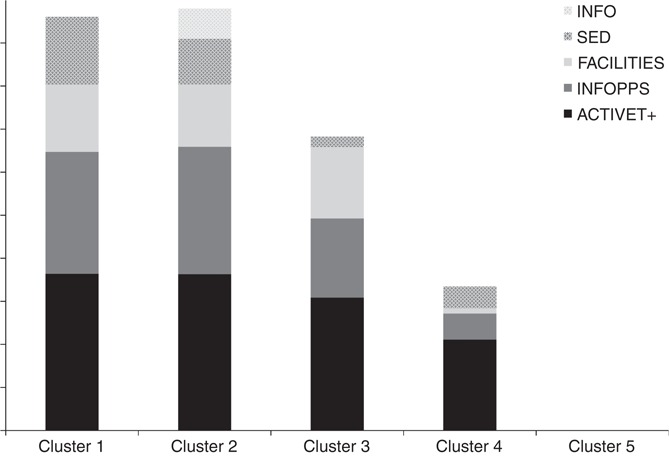
Stacked bar chart showing the distribution of factors ACTIVET+, INFOPPS, FACILITIES, SED and INFO amongst the five clusters of worksites

**Figure 2:**
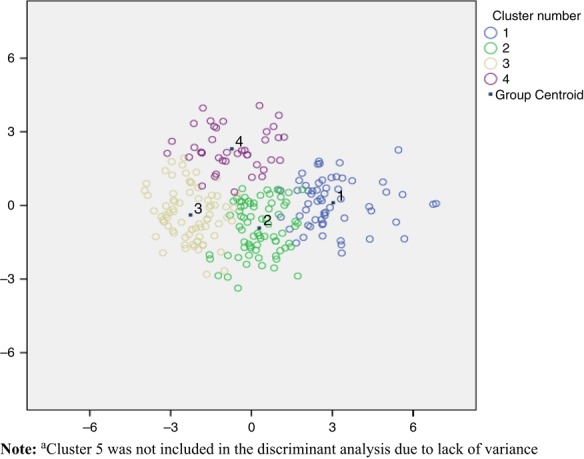
Scatter plot summarising the separation of clusters in the discriminant model

Five factors were identified to be present in the Worksite Health Promotion Survey data. The items belonging to each factor are provided in Table III. In summary, factor 1 was characterised by the most structures many of which were geared towards supporting active travel (e.g. financial incentives to actively commute) and informational/promotional approaches, support and policies aimed at accessing physical activity opportunities in the local area (ACTIVET+). Factor 2 was characterised by the provision of information on physical activity and opportunities to experience physical activity both within and outside of the workplace (INFOPPS). Factor 3 was characterised by the presence of onsite physical activity structures such as facilities and classes (FACILITIES). Factor 4 was characterised by structures aimed at reducing sedentary behaviour (e.g. standing breaks; SED). Factor 5 was characterised by the sole provision of information on physical activity outside of the workplace (INFO). From this, five clusters of worksites emerged. The contribution of the five factors to these clusters is shown in Table IV and presented graphically in Figure 1. In total, 23.6 per cent (*n*=139) of worksites (cluster 1) provided the most support structures, the majority of them being the provision of information on opportunities inside and outside of the workplace (INFOPPS) and structures to reduce sedentary behaviour (SED), followed by structures relating to active travel and access to local opportunities (ACTIVET+) and onsite facilities/classes (FACILITIES). In total, 17.3 per cent (*n*=102) of worksites (cluster 2) also provided a large number of supports for physical activity, however, for the majority of these worksites these were all related to the provision of information on opportunities outside of the workplace (INFO). Some of these worksites also provided information on opportunities inside the workplace (INFOPPS) and on active travel (ACTIVET+). 24.1 per cent (*n*=142) of worksites (cluster 3) provided fewer supports in general. They generally reported having onsite facilities/classes (FACILITIES) and some had structures relating to active travel and accessing local opportunities (ACTIVET+), but nothing more. In total, 12.2 per cent (*n*=72) of worksites (cluster 4) provided very few supports, with only a small amount of structures relating to active travel and access to local opportunities (ACTIVET+). In total, 22.6 per cent (*n*=133) of worksites (cluster 5) did not provide any support structures for physical activity. K-means analysis replicated the patterns of the clusters in all cases and suggested that optimal clustering had been achieved. Figure 2 presents the clusters based on discriminant analysis. Pooled within-group correlations suggested that multicollinearity was not a problem as covariance coefficients were less than 0.8. The Wilks Lamba test and *χ*^2^ statistics confirmed that all five factors significantly contributed to participating worksites cluster membership (*p*<0.05).

### Associations between workplace factors and physical activity variables

Promoting active travel and providing information on the opportunities to be active in the local area (ACTIVET+) was associated with a higher frequency of active commuting (*β*=0.84, *p*<0.05), but also with being more sedentary at work (*β*=−0.32, *p*<0.001). Provision of information on physical activity and opportunities to experience physical activity both within and outside of the workplace (INFOPPS) was associated with being less sedentary and more active at work (*β*=0.14, *p*<0.001), but also with a lower frequency of active commuting (*β*=−0.13, *p*<0.001). The presence of onsite facilities and classes (FACILITIES) was associated with being less sedentary and more active at work (*β*=0.17, *p*<0.001) and with meeting MVPA guidelines (*β*=0.05, *p*<0.05). Structures which were aimed at reducing sedentary behaviour (SED) were associated with meeting MVPA guidelines (*β*=0.08, *p*<0.05) and having a higher frequency of active commuting (*β*=0.13, *p*<0.05), but also being more sedentary at work (*β*=−0.16, *p*<0.01). The sole provision of information on physical activity outside of the workplace (INFO) was associated with being less sedentary and more active at work (*β*=0.60, *p*<0.001). These associations are summarised in Table V.

## Discussion

The present study identifies five profiles related to the promotion of physical activity by workplaces across England. Two clusters (40.9 per cent) promoted physical activity relatively extensively. Two clusters (36.3 per cent) engaged in relatively minimal promotion and one cluster which contained 22.6 per cent of the sample did not have any of the 50 suggested items in place to promote physical activity. The most popular policies and practices were around active travel and reducing sedentary behaviour with information on physical activity outside the workplace also being regularly disseminated. Very few of the workplaces provided onsite facilities or classes to support physical activity.

It may be a challenge for many organisations to know which structures to introduce to their worksite which will have the greatest chance of positively impacting their workforce. Further, it may be a challenge for other stakeholders to know which efficacious structures have the greatest chance of being adopted and maintained by those organisations. Uncovering what is commonly utilised provides a strong indication of what is acceptable to workplaces and therefore feasible as a potential interventional strategy. The present findings may be helpful in identifying the types of structures which may hold the greatest potential to influence employee behaviour. We found mixed results to support the popular practices and policies encouraging active travel and reduced sedentary behaviour. Whilst structures targeting active travel were associated with a greater frequency of actively commuting to and from the workplace, they were not associated with meeting MVPA guidelines. It is unclear whether this could be a result of commuting journeys being too short or light in intensity to count as “guideline-fulfilling” MVPA or whether these adults substitute other forms of physical activity with active commuting. Indeed, these employees were also less likely to be active and more likely to be sedentary whilst at work. It is possible that employees who actively commute compensate by being less active at work. Research by Buehler *et al.* (2011) shows that increases in active commuting can be translated into gains in overall MVPA, however, national promotional efforts have been more successful at increasing the proportion of people participating and not at increasing the volume of active travel at an individual level. Workplaces may therefore need to use additional policies and practices alongside ones geared towards active travel if the aim is to increase overall MVPA of individual employees.

On the other hand, whilst structures promoting reduced sedentary behaviour were surprisingly associated with being less active and more sedentary whilst at work, they were also associated with more frequent active commuting behaviour and with meeting MVPA guidelines. These are interesting findings. It is possible that such structures have been introduced into workplaces which require employees to engage in a large amount of sedentary behaviour and so have encouraged the substitution of sedentary behaviour with MVPA outside of the workplace, for instance, walking to the shops rather than driving. An earlier study by Crespo *et al.* (2011) similarly found that American employees who reported having more strategies supporting physical activity in their workplace were more likely to be recreationally active but were less active at work and were more sedentary overall. It is possible that workers who perceive their employer to support physical activity generally may feel moved to be more active, regardless of the way in which physical activity is promoted.

It is perhaps surprising that workplaces from only two clusters were characterised by disseminating information regarding physical activity within or outside the workplace as this could be considered a relatively low-cost option for promoting physical activity, especially if the workplace already has a website/intranet, newsletter, etc. in place for general purposes. In this study, employees who reported receiving information from their workplace regarding physical activity tended to be more active in their workplace but were not more likely to meet MVPA guidelines. It is possible that some of these are results of message prompts, e.g. to take the stairs, contributing to sporadic activity within the workplace which lacks the continuity of guideline-fulfilling MVPA and is also difficult to enact in other settings (Webb and Smith, 2011).

Onsite provision of physical activity opportunities in the workplace overcomes many of the barriers to physical activity cited in previous research (Bauman *et al.*, 2012). Only one cluster of workplaces was characterised by the presence of onsite physical activity facilities (e.g. changing facilities and sports or gym equipment) and classes suggesting that this is not a preferred option for workplaces to promote physical activity. The cost of providing such facilities and opportunities may explain why employees report this kind of provision in only 24.1 per cent of workplaces. In contrast, this study found strong evidence to support the provision of facilities and classes in workplaces as they were associated with more activity whilst at work and with meeting MVPA guidelines. This suggests that it may be necessary to encourage and assist employers in improving the physical environment of the workplace to promote increased physical activity. However, it may be possible that some employees do not want to engage in physical activity during working hours and their needs and interests should be assessed prior to investing in facilities. One strategy to overcome the barriers of costs, providing onsite facilities and employee interests in physical activity at work could be to communicate the health benefits of being physically active to employees and the potential economic benefits of a healthy and active workforce to employers. Further, in instances when the cost barrier cannot be overcome, workplaces should be encouraged to signpost employees to local activities and facilities.

The results of this study are associative only and so must be interpreted with caution. Further, data were collected through self-report and so measurement error may have influenced the results. We achieved a response rate of only 30 per cent to the Worksite Health Survey. Online surveys typically attain lower response rates than other modes (Fan and Yan, 2010); however, further study is needed to test the generalisability of the findings. A next step could be to investigate whether certain structures are more acceptable and/or more effective within worksites with certain characteristics. However, this is the first study to investigate the types of policies and practices employed by a large sample of workplaces across England and to provide some guidance on which may have the greatest association with the physical activity of employees. Workplace health promotion is often limited by the resources available to a given workplace in support of its various dimensions. It is important to encourage further exploration of existing practices with regards to physical activity promotion to limit the wasting of resources and support the development of effective, acceptable and feasible workplace interventions.

## Conclusions

Five strategies for physical activity promotion within the workplace emerged from the present research. These included structures broadly described as targeting active travel, information about physical activity outside the workplace, facilities and onsite opportunities, sedentary behaviour and information about physical activity within the workplace. Five common profiles of promoting physical activity in 308 worksites across England were also identified. This research could be used to advise worksites with limited resources dedicated specifically for employee physical activity on the likely best course of action. Where feasible, facilities and classes should be provided onsite as this was associated with the most positive behavioural outcomes. The more common practices of promoting active travel or reduced sedentary behaviour were associated with some increases in physical activity but additional strategies may be needed to increase the proportion of the workforce meeting MVPA guidelines.

## Figures and Tables

**Table I:** Demographic characteristics of respondents to the workplace challenge survey

	*n*=3,360	
Characteristic	*n*	%
*Gender*		
Male	1,117	33.2
Female	2,243	66.8
*Age, years*		
Mean ± 95% CI	39.5	39.1, 39.9
*Ethnic group*		
White British	3,175	95.4
Black/Black British	57	1.7
Asian/Asian British	65	2.0
Mixed	31	0.9
*Highest educational qualification*		
Degree	2,150	64.0
Business and Technology Education Council (BTEC) Higher/Advanced Level (A Level)	691	20.6
BTEC National/General Certificate of Secondary Education (GCSE)	441	13.1
None/other	78	2.3
*Job type*		
Managerial, e.g. office manager, finance manager	947	29.5
Professional, e.g. nurse, teacher, police officer	1,197	37.2
Clerical/Admin, e.g. secretary, office worker	965	30.0
Manual/Technical, e.g. postal worker, farm worker	104	3.2
*General health*		
Excellent/Good	2,683	79.9
Fair/Poor	677	20.1
*Activity status at work*		
Sedentary	2,880	85.7
Active	480	14.3
*Physical activity engagement*		
Meets guidelines (⩾150 mins/wk)	890	26.5
Does not meet guidelines (<150 mins/wk)	2,470	73.5
*Frequency of active commuting (trips/wk)*		
Mean ± 95% CI	2.6	2.4, 2.7

**Table II:** Demographic characteristics of respondents to the worksite health promotion survey

	*n*=588	
Characteristic	*n*	%
*Gender*		
Male	173	30.7
Female	391	69.3
*Age*		
16-24	39	6.8
25-34	176	30.7
35-44	152	26.5
45-54	147	25.7
55+	59	10.3
*Worksite type*		
Private	90	16.9
Statutory	214	40.2
Voluntary	5	0.9
Charity	59	11.1
Other	165	30.6
*Number of sites*		
Single site	105	19.7
Multi-site	429	80.3
*Location*		
City/Town	385	72.1
Urban	59	11.0
Rural	59	11.0
Other	31	5.8
*Number of employees*		
<50	63	11.8
51-250	105	19.7
251-1,000	141	26.5
1,001-3,000	89	16.7
>3,000	134	23.1
*Proportion of employees in office-based sedentary jobs (%)*		
<10	38	7.3
10-25	66	12.7
26-50	118	22.8
51-75	169	32.6
76-99	102	19.7
100	25	4.8

**Table III:** Self-reported items relating to worksite promotion of physical activity and the factor they relate to

	Factor 1 – active travel and local opportunities (ACTIVET+)	Factor 2 – provision of information and opportunities within and outside the workplace (INFOPPS)	Factor 3 – facilities and onsite classes (FACILITIES)	Factor 4 – structures to reduce sedentary behaviour (SED)	Factor 5 – provision of information (INFO)
Have flexible working policies	0.596^a^	0.024	0.273	0.447	0.021
Have incentive schemes for active travel	0.724^a^	0.378	−0.090	0.014	0.153
Have sustainable travel/active travel policies	0.753^a^	0.094	0.023	0.045	0.087
Offer reduced price gym memberships	0.695^a^	−0.079	0.230	−0.261	−0.251
Have walking/cycling routes/maps	0.779^a^	0.081	0.055	−0.074	−0.013
Disseminate written information on how to be more active	0.726^a^	0.314	−0.087	−0.227	0.298
Use websites/e-mail to inform employees about physical activity opportunities	0.798^a^	0.128	−0.000	−0.054	0.191
Use online materials to promote physical activity guidelines and/or the benefits of being active	0.745^a^	−0.076	−0.031	−0.073	0.069
Offer subsidised corporate memberships at local facilities	0.633^a^	0.202	0.027	0.299	−0.039
Have bicycle rack facilities	0.739^a^	−0.012	0.001	0.255	0.024
Offer tailored opportunities to be physically active both within and outside the workplace	−0.015	0.815^a^	−0.017	0.088	0.045
Use newsletters and staff/team meetings inform staff about physical activity opportunities	0.130	0.754^a^	0.091	0.256	0.184
Promote the physical activity guidelines and/or the benefits of being active through team meetings, bulletin boards, e-mails and seminars	0.145	0.774^a^	0.116	0.050	0.003
Offer team activity challenges	0.198	0.615^a^	0.175	0.362	−0.107
Offer lunchtime activity groups	0.158	0.807^a^	0.046	−0.075	−0.024
Offer onsite activity taster sessions	0.075	0.852^a^	0.093	0.015	0.068
Offer talks and presentations on physical activity	0.124	0.704^a^	0.435	−0.037	−0.007
Offer taster sessions at local leisure facilities	0.106	0.599^a^	0.093	−0.199	−0.037
Deliver physical activities to employees through outside organisations	0.041	0.638^a^	0.241	0.031	0.031
Offer company leagues, ladders and competitions	−0.011	0.700^a^	0.129	0.016	−0.003
Offer sport or activity clubs	0.088	0.781^a^	0.139	0.021	0.004
Provide changing rooms	0.359	−0.124	0.624^a^	−0.002	−0.174
Provide sports equipment	0.333	−0.100	0.717^a^	0.374	0.008
Provide an onsite gym	−0.030	0.232	0.749^a^	0.070	0.036
Provide lockers	0.152	−0.146	0.738^a^	−0.029	0.059
Provide showers	−0.263	0.244	0.567^a^	0.032	0.132
Offer onsite activity classes	−0.044	0.390	0.660^a^	0.225	0.046
Have organisational policies and practices to encourage breaks from prolonged sitting	0.386	0.140	0.266	0.501^a^	0.182
Encourage employees to break up their sitting time during the working day	0.332	0.228	0.192	0.615^a^	0.115
Encourage employees to get away from their desks at lunchtime	0.104	0.286	0.057	0.774^a^	0.097
Have policies on breaking up sitting time during the working day	−0.146	0.013	−0.009	0.842^a^	0.079
Hold walking meetings	0.083	0.158	0.087	0.640^a^	−0.075
Use bulletin boards to inform employees about physical activity opportunities	0.174	0.075	0.085	0.093	0.715^a^
Use newsletters to promote the physical activity guidelines and/or the benefits of being active	0.001	0.124	0.104	0.035	0.730^a^

**Note:** ^a^Factors relating to self-reported items

**Table IV:** Factor loading results from the cluster analysis

Workplaces and the types of structures used to promote physical activity					
Factor	Cluster 1 (23.6%)	Cluster 2 (17.3%)	Cluster 3 (24.1%)	Cluster 4 (12.2%)	Cluster 5 (22.6%)
ACTIVET	*(✓) 0.58*	*(✓) 0.60*	*(✓) 0.62*	*(✓) 0.59*	0
INFOPPS	*✓ 0.71*	*(✓) 0.48*	0.12	0.20	0
FACILITIES	*(✓)*^*a*^ *0.58*	0.04	*✓ 0.72*	0.02	0
SED	*✓ 0.63*	0.13	0.09	0.19	0
INFO	0.28	*✓ 0.82*	0.27	0.28	0

**Notes:** Cluster means are given from *K*-means analysis. ✓ denotes strong loading; (✓) denotes some loading. ^a^Strong loading using *K*-means analysis

**Table V:** Factor association with meeting MVPA guidelines, activity status at work and active commuting

Factor	Meeting MVPA guidelines	Activity status at work^a^	Active commuting
ACTIVET	0.986 (0.95, 1.03)	0.727 (0.69, 0.77)*	0.052 (0.02, 0.15)*
INFOPPS	0.967 (0.93, 1.00)	1.147 (1.09, 1.21)*	−0.09 (−0.19, −0.07)*
FACILITIES	1.052 (1.01, 1.10)*	1.183 (1.10, 1.27)*	0.032 (−0.01, 0.15)
SED	1.080 (1.01, 1.15)*	0.852 (0.77, 0.94)*	0.042 (0.01, 0.24)*
INFO	0.982 (0.85, 1.14)	1.829 (1.49, 2.25)	0.027 (−0.07, 0.45)

**Notes:** ^a^Regression predicted the likelihood of reporting job to be mainly “manual” or “very manual” as opposed to “sedentary” or “standing”. *p<0.05
